# Metal-Free Prosthodontic Treatment of Periungual Allergic Contact Dermatitis: A Case Report

**DOI:** 10.7759/cureus.83598

**Published:** 2025-05-06

**Authors:** Yasuda Hiroyasu, Ito Kenji, Matsuda Yuko, Fukushima Ryo, Akita Daisuke

**Affiliations:** 1 Department of Partial Denture Prosthodontics, Nihon University School of Dentistry, Tokyo, JPN; 2 Department of Oral and Maxillofacial Surgery Ⅱ, Nihon University School of Dentistry, Tokyo, JPN

**Keywords:** allergic contact dermatitis, case report, metal allergy, patch testing, prosthodontics

## Abstract

Prosthodontic treatments utilize various procedures and materials such as gold, silver, copper, palladium, cobalt, chromium, and titanium to restore oral function; however, these materials are known to induce allergic contact dermatitis (ACD). We present herein a case in which a patient showed improvement in ACD after undergoing prosthetic treatment using metal-free dental materials. A 53-year-old female patient with periungual inflammation was referred to our department due to instability with her maxillary prosthesis, which was attributed to severe peri-implantitis. Skin tests were conducted due to the suspicions of a metal allergy, and patch testing revealed positive reactions to dental metals, specifically cobalt and palladium. As a result of this finding, all of the patient’s metal-based prostheses were systematically replaced with provisional crowns. During this period, any teeth and dental implants affected by severe periodontitis were extracted, after which the patient was fitted with zirconia-based prostheses, resulting in a significant reduction in periungual inflammation. In this case, therefore, the utilization of metal-free prostheses facilitated improvement of the patient’s ACD, providing valuable insights for dental practitioners managing patients with ACD.

## Introduction

The primary objective of prosthodontic treatment is to restore oral function following tooth loss due to periodontal diseases, dental caries, root fractures, maxillofacial trauma, cysts, or tumors [1]. Although a range of metallic and organic materials are utilized in the fabrication of dental prostheses, some of these materials are known to have allergenic properties capable of inducing symptoms inside and outside the oral cavity [2]. As contact allergies frequently follow the process of sensitization that occurs on the skin, they commonly affect the hands. Allergic reactions involving the skin often present as eczema, contact dermatitis, or plantar-palmar rashes, among other manifestations [3]. It has been suggested that mercury, nickel, chromium, palladium, and cobalt in dental metals cause allergic contact dermatitis (ACD) [4], with several studies naming nickel as the most frequently reported contact allergen [2,5]. 

Dental metal allergy pertains to the manifestation of ACD, which is believed to be linked to the utilization of metal alloys in dental procedures. Nonetheless, this association does not inherently imply a causal relationship. Therefore, the prosthodontic treatment of patients with ACD can present significant challenges. There has been growing interest in the utilization of metal-free prostheses owing to their superior aesthetic qualities, biocompatibility, and durability, positioning them as viable alternatives to traditional metal prostheses [6]. In particular, zirconia-based dental ceramics exhibit enhanced mechanical strength compared to conventional glass-ceramic restorations [7]. We present herein the case of a female patient who showed improved periungual ACD after her metal prostheses were removed based on positive patch tests and replaced with a zirconia-based metal-free prosthesis.

## Case presentation

A 53-year-old woman was referred to the Dental Hospital at Nihon University School of Dentistry due to the loosening of fixed implants in her maxilla, which had been installed by a different dental clinic. The patient reported that these implants were placed approximately 10-15 years prior at the aforementioned clinic, and there were no notable abnormalities in the oral cavity until a few days prior to presentation, at which point the dental implant began to exhibit instability. The patient's palms and fingers were overall rough and irritated, in an eczema-like state, and the nails were cracked. The patient was a smoker with allergies to metals and shellfish, but otherwise she was in good physical condition with no bleeding disorders.

**Figure 1:**
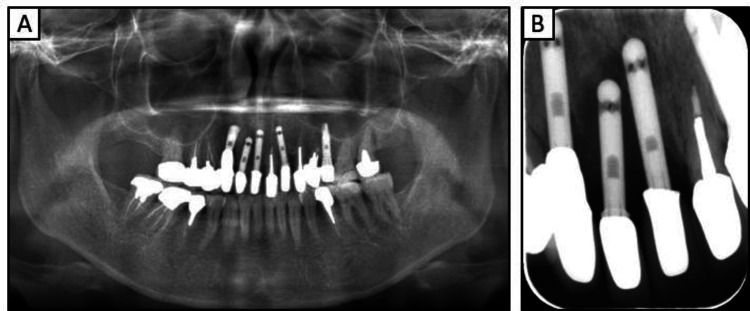
Preoperative radiographs obtained at the patient’s initial visit (A)  Panoramic radiograph (B)  Radiograph of the right anterior maxilla

Extra- and intraoral examinations revealed no facial asymmetry or abnormalities of the temporomandibular joint and no abnormalities in the frenula attachment or the mucous membranes covering the residual ridge, respectively; however, the lips were rough and inflamed. The missing teeth in the upper jaw were the 17, 13, 12, 11, 22, and 25. Except for tooth 17, tooth loss of the maxilla was treated using dental implant prosthetics. The probing depth associated with the dental implant located in section 22 measured approximately 9 mm, and signs of mobility and purulent discharge were noted. Additionally, upper teeth 15, 14, and 27 were non-vital, with an inadequate filling in the root canal filling of tooth 27. The probing depths of teeth 16, 15, 14, 21, 23, 24, and 26 were approximately 2-3 mm with no pathological mobility observed; however, the probing depth and mobility of tooth 27 were approximately 7 mm and grade I, respectively. Mandibular teeth 47, 46, 45, and 35 were non-vital teeth with root canal fillings, and the probing depth of all mandibular teeth was approximately 2-3 mm, with no pathological mobility observed. The patient’s occlusion was stable. A radiographic examination revealed a significantly high absorption around the implants in the anterior maxilla (Figure 1).

The patient initially expressed apprehension about the mobility of her implant; however, given the reported metal allergy, we advised the patient to undergo patch testing (Table 1). The patient agreed to the patch testing and subsequently consented to the removal of anterior dental implants affected by peri-implantitis, extraction of tooth 27, removal of the metal prosthesis, and subsequent prosthetic treatment using non-metal materials.

**Table 1:** Result of patch testing The patch test was performed on the patient's back. The evaluation of the patch test was carried out in accordance with the criteria established by the International Contact Dermatitis Research Group (ICDRG), which categorizes reactions as follows: "-" indicating no reaction; "?+" signifying weak erythema; "+" representing erythema accompanied by infiltration and papules; "++" denoting erythema with infiltration, papules, and small blisters; and "+++" indicating the presence of large blisters Patch testing revealed a positive reaction for dental metals such as cobalt, palladium

	Type	After 72 hours	After one week	After two weeks
1	Nickel	+	++	+
2	Tin	-	-	-
3	Chromium	-	-	-
4	Cobalt	-	-	+
5	Manganese	-	-	-
6	Mercury	-	-	-
7	Gold	-	-	-
8	Platinum	-	-	-
9	Silver	-	-	-
10	Palladium	-	+	+
11	Indium	-	-	-
12	Vaseline	-	-	-
13	Zinc	?+	?+	-
14	Aluminum	-	-	-
15	Copper	-	-	-
16	Iridium	-	-	+
17	Iron	-	-	-

Clinical procedure

While awaiting the results of the patch testing, the dental implant in tooth 12 became dislodged. Consequently, we implemented a temporary solution using restorative dental materials (Unifil Loflo Plus; GC Dental Co. Ltd., Tokyo, Japan). The patch test results were positive for several dental metals, specifically cobalt and palladium (Table 1). Based on these results, we informed the patient that prosthetic treatment using a metal-based fixed or removable partial denture was not feasible. She subsequently expressed the desire to extract the implants located in the upper anterior region and opted for treatment with metal-free fixed partial dentures. 

**Figure 2:**
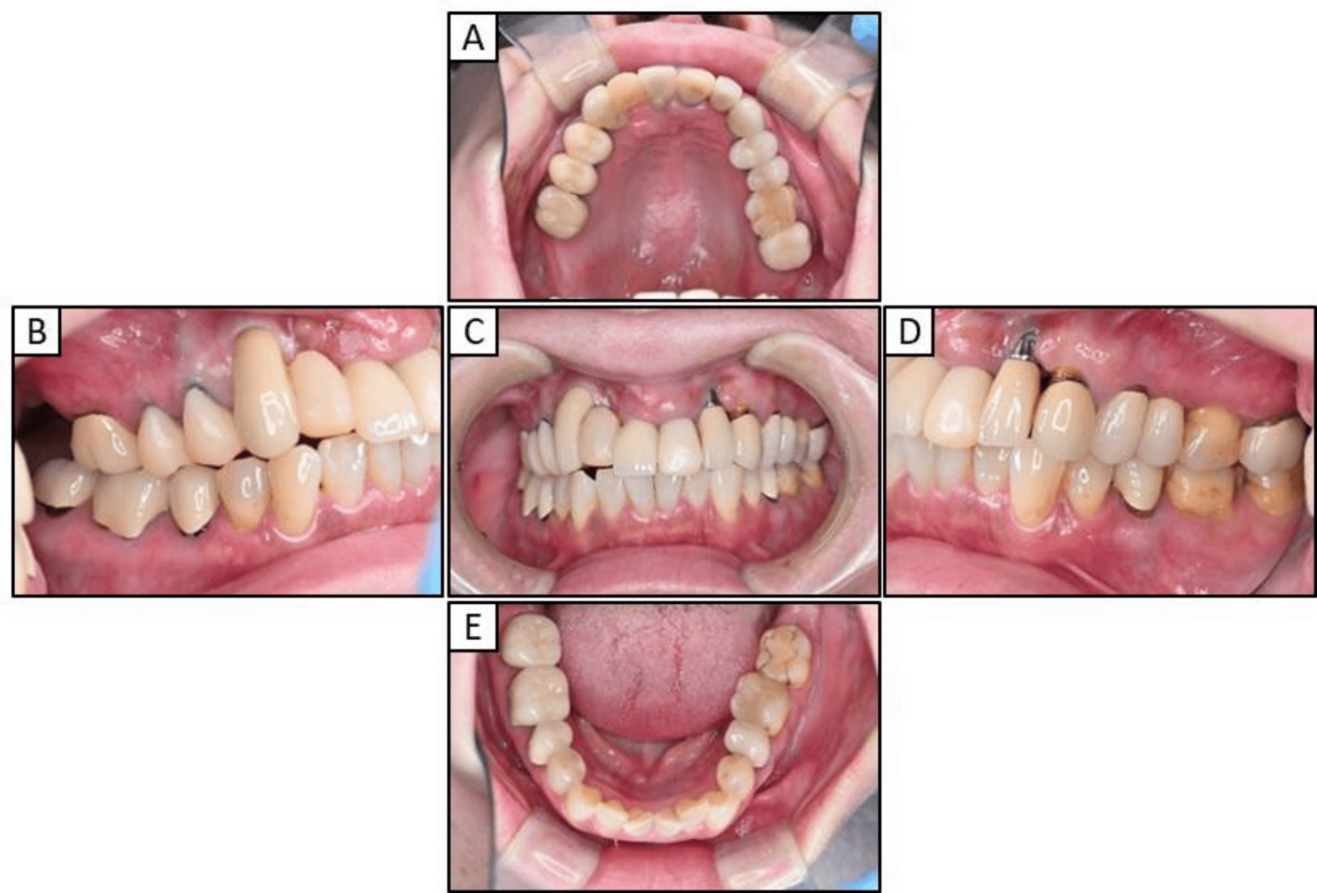
Intraoral photographs obtained early in the treatment course (A) Maxillary occlusal view (B) Right lateral view (C) Frontal view (D) Left lateral view (E)  Mandibular occlusal view

**Figure 3:**
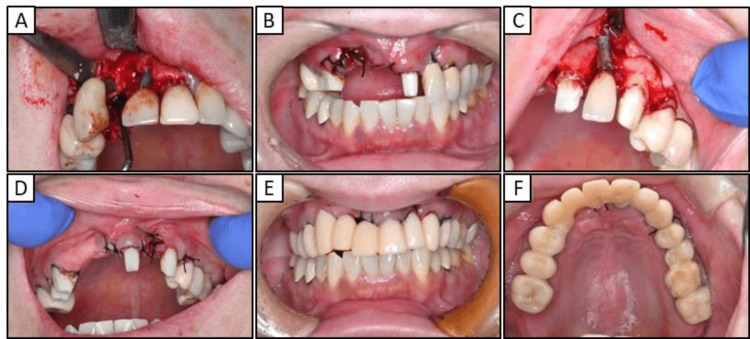
Intraoral photographs obtained during maxillary treatment (A) Implant extraction on the right side (B) Post-right maxillary implant extraction intraoral photograph (C) Implant extraction on the left side (D) Post-left maxillary implant extraction intraoral photograph (E) Frontal view of provisional crown after extracting the implants (F) Maxillary occlusal view of eth provisional crown after post-implant extraction

First, we replaced the right premolar area and tooth 21 with a temporary crown (Figure 2) and then extracted the implants in teeth 13 and 11 (Figures 3A, 3B). After replacing the metal-based prostheses on the upper left side with a temporary crown, the implant in tooth 23 was extracted (Figures 3C, 3D). The metal prostheses in the upper jaw were then replaced with temporary resin-based crowns (Figures 3E, 3F). While the extraction site was healing, the porcelain-fused-to-metal crowns on teeth 35, 45, 46, and 47 were replaced with temporary crowns until the zirconia prostheses were placed. Tooth 27 was extracted due to chronic purulent apical periodontitis.

**Figure 4:**
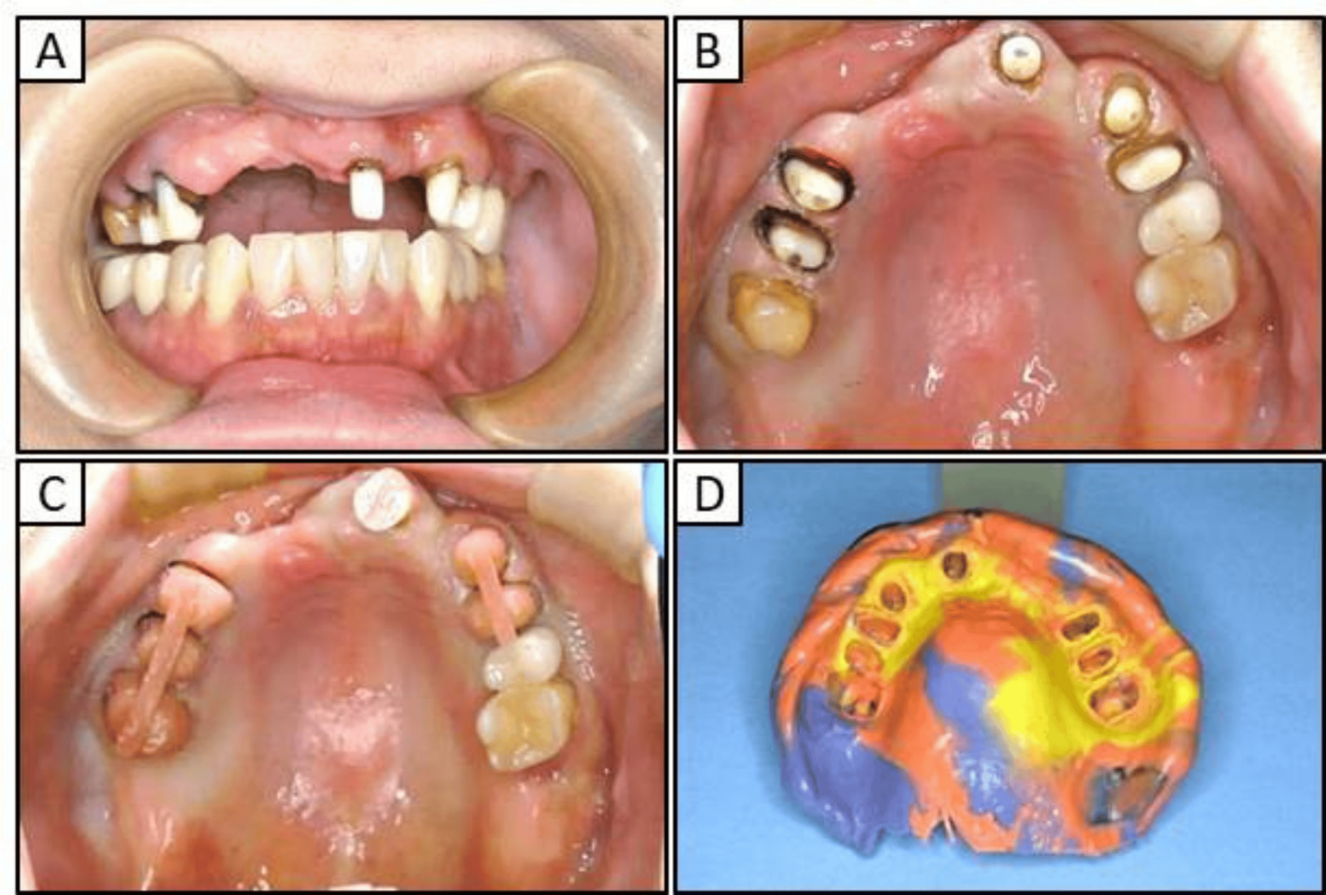
Images obtained prior to manufacturing the maxillary definitive prosthesis (A) Front view of the maxillary abutment teeth (B) Maxillary occlusal view of the abutment teeth (C) Maxillary occlusal view using individual trays for abutment impressions (D) Impression using individual trays for abutment impressions, an individual tray, and three kinds of silicone impression materials

After observing the recovery of oral function, including esthetics, speech, and comfort, over the course of several months, a final prosthetic impression of the upper jaw was made using individual trays for abutment impressions, individual trays, and three silicone impression materials (Figure 4) (EXAHIFLEX; GC Dental Co., Ltd. and SILDE FIT; SHOFU INC., Kyoto, Japan).

**Figure 5:**
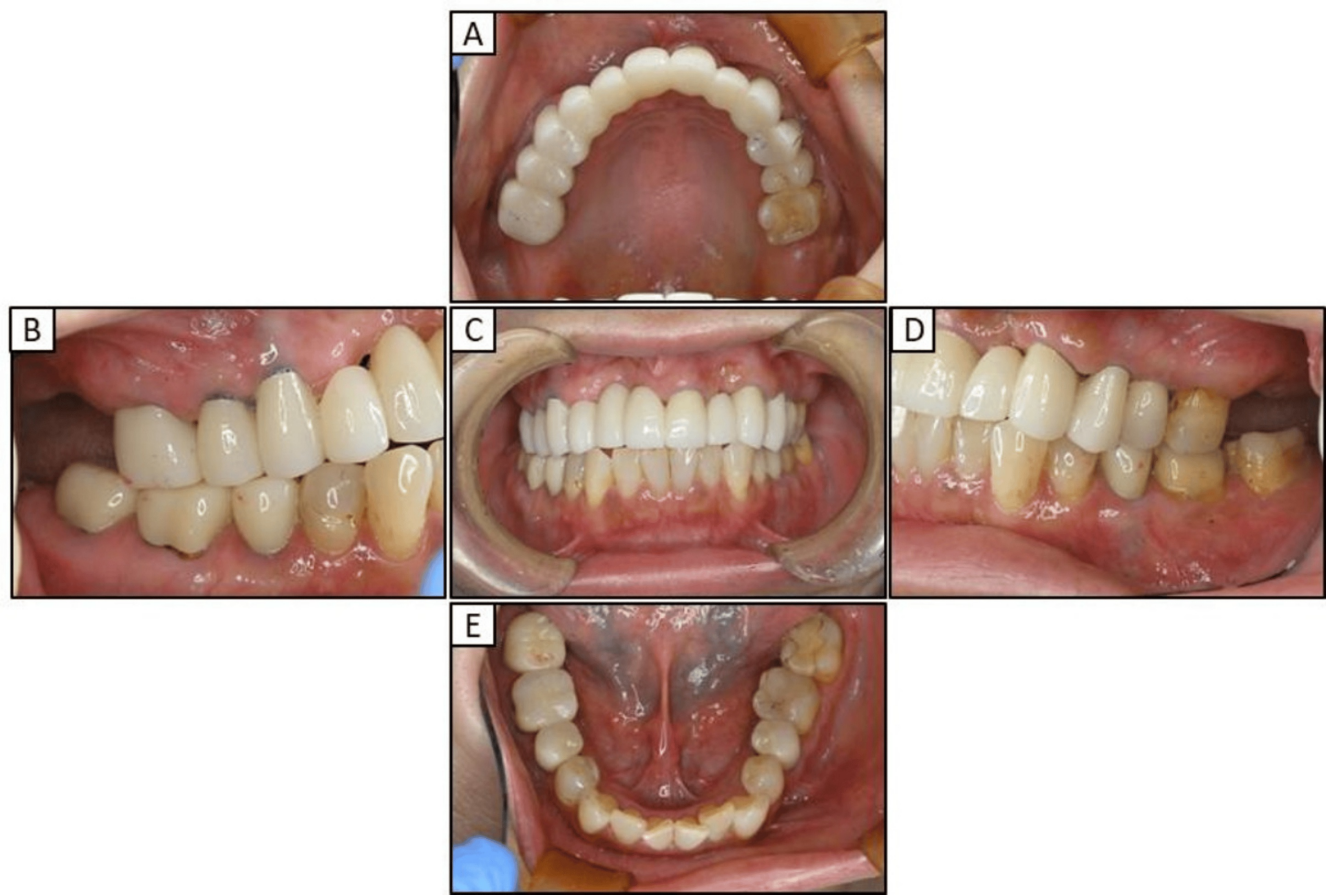
Postoperative intraoral photographs (A) Maxillary occlusal view (B) Right lateral view (C) Frontal view (D) Left lateral view (E) Mandibular occlusal view

**Figure 6:**
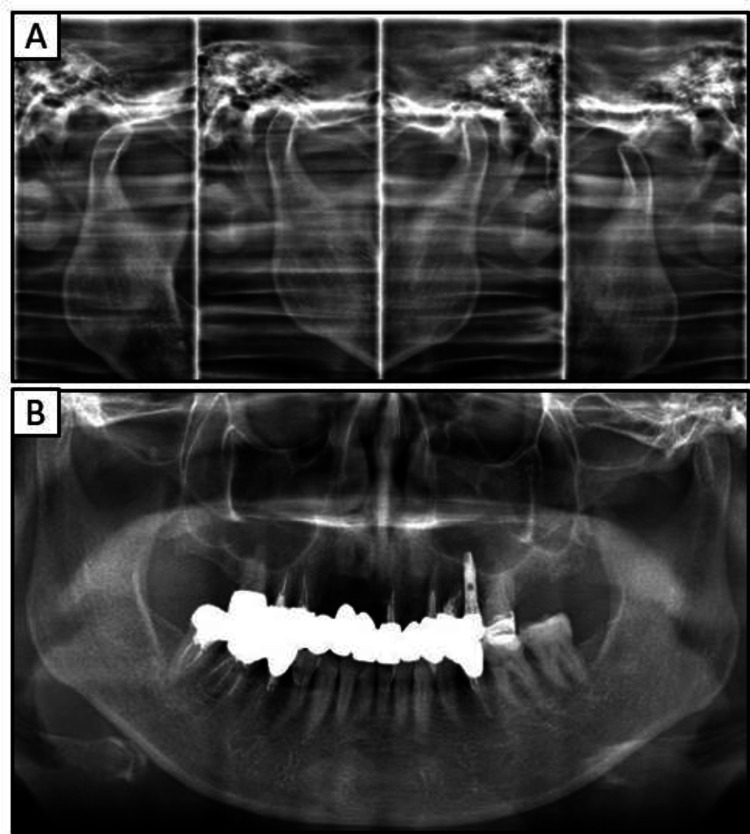
Postoperative radiographs (A) Radiographic image of temporomandibular joint (B) Panoramic radiograph

**Figure 7:**
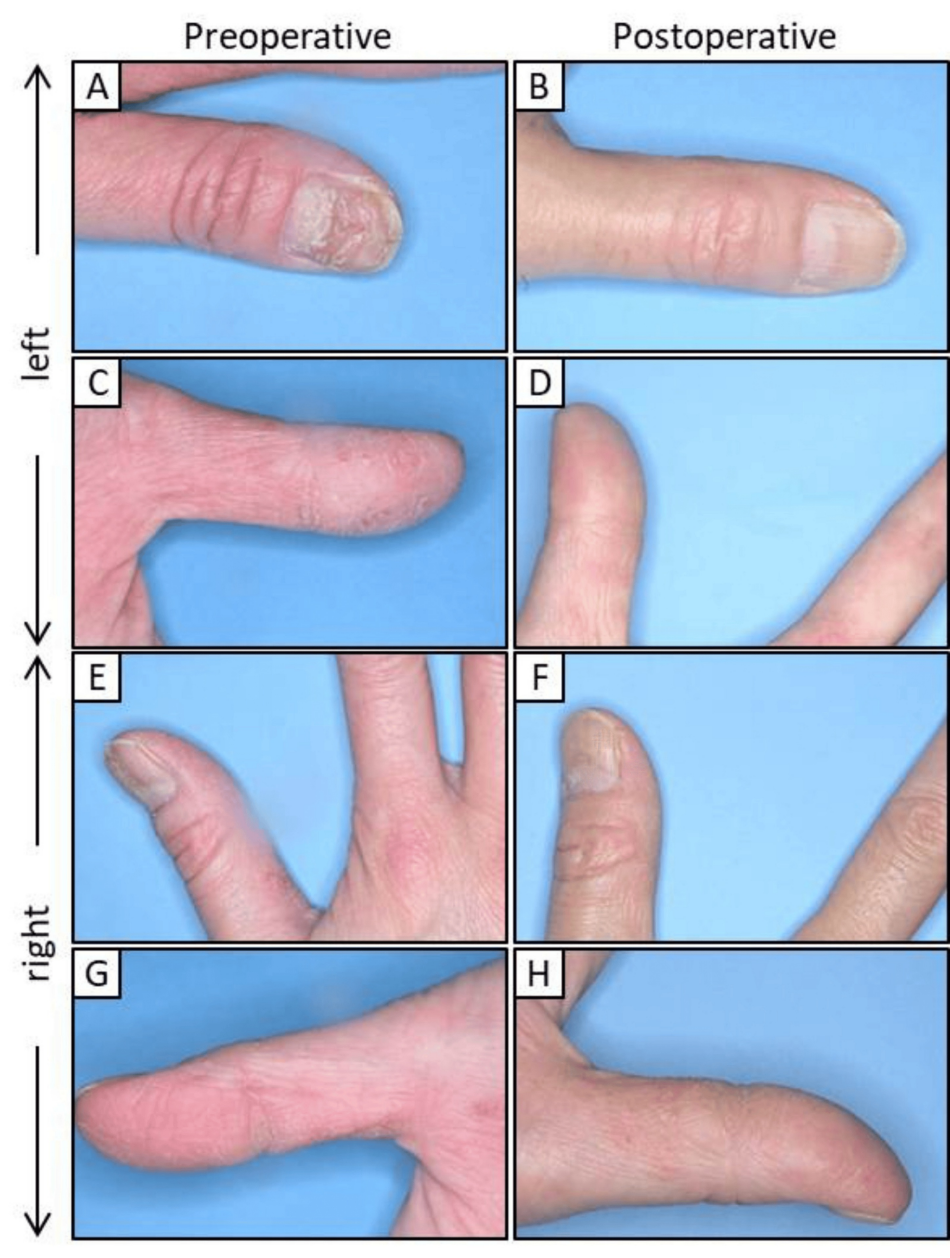
Pre- and postoperative photographs of the fingers (A–D) Left hand (E–H) Right hand

By approximately 30 months after the initial consultation, all the metal prostheses were replaced with zirconia-based prostheses, eliminating the use of metals (Figure 5). Radiographic evaluations did not reveal any atypical findings within the oral cavity or temporomandibular joints (Figure 6). Figure 7 shows the pre- and post-treatment images of the patient's fingers. On the left hand, the ACD in the periungual region showed improvement (Figures 7A-7D); however, no significant changes were observed on the right hand (Figures 7E-7H).

## Discussion

Metal allergies are commonly classified as contact- or delayed-type hypersensitivities. Clinical delayed-type hypersensitivity reactions may present as localized contact dermatitis at the site of antigen exposure on the skin or as systemic contact dermatitis, wherein the antigen is disseminated throughout the body via the bloodstream in sensitized individuals, leading to effects on distant cutaneous regions [8]. Dental metal allergies refer to hypersensitivity reactions or sensitization attributed to metal elements utilized within the oral cavity in dental practices. The underlying mechanisms that contribute to the development of dental metal allergies remain poorly understood, although it is known that they arise through sensitization and elicitation. Sensitization refers to the initial phase in which the immune system recognizes and retains the memory of an antigen, which occurs when antigen-presenting cells capture the antigen and subsequently present it to T cells, which then encode relevant information. Following sensitization and upon re-exposure to the previously encountered antigen, T cells specific to that antigen migrate to and infiltrate the site of contact, resulting in an inflammatory response known as the elicitation phase [9]. Patch testing leverages these immunological mechanisms to intentionally provoke allergic reactions and is a valuable tool for identifying metals that induce sensitization [8,10].

The primary etiology of dental metal allergies is thought to stem from the leaching of metals from the materials utilized within the oral cavity. Animal models and cellular studies have indicated that ionized metals may elicit immunological responses through their interactions with proteins [11,12]. The predominant clinical manifestations associated with dental metal allergies include contact dermatitis and mucositis localized to the area of metal contact, in addition to eczema-like reactions and palmoplantar pustulosis occurring at remote sites.

Several studies have shown that ACD is more prevalent in women [13,14]. Furthermore, more than half of patients with a sensitivity to nickel also showed sensitivity to at least one other metal [15]. The elevated incidence of nickel hypersensitivity observed in women may be attributed to environmental exposure, as women typically wear jewelry, earrings, and other metal products more frequently and from an earlier age [16]. Research indicates that prevalent locations for ACD are the fingers, hands, head, and neck [17]. The precise causal relationship between ACD and metal allergies remains unclear; however, certain forms of dermatitis are associated with periodontal disease and apical periodontitis [18-20]. Evidence has suggested that addressing chronic inflammation and removing the dental metals utilized in prosthetic devices may improve dermatological conditions [11].

In the current case of the 53-year-old woman who presented to our hospital with a chief complaint of peri-implantitis affecting the anterior maxillary teeth (Figure 1) and roughness on her fingers and hands, the patient did not exhibit any mucosal conditions, such as lichen planus, within the oral cavity; however, cheilitis was noted during the initial examination. Based on these symptoms and a reported allergy, patch testing was performed, which revealed positive reactions to dental metals, including nickel (Table 1). In response to these results, the treatment plan necessitated a comprehensive evaluation of the entire oral cavity (Figure 2). The primary goal of prosthetic treatment is the restoration of oral function; however, the choice of materials for dental prosthetics needs a meticulous evaluation of their mechanical strength and aesthetic characteristics. This choice is critical because it substantially affects the longevity of the restoration and dependability of the treatment results [6]. As the patient in the current report tested positive for multiple dental metals, such as cobalt and palladium, in addition to nickel, the use of dental metals for prosthetic treatment was not recommended. Owing to their enhanced aesthetic qualities, biocompatibility, and durability, ceramic systems represent an excellent alternative for crown restorations and are being increasingly utilized with good efficacy in dental rehabilitation [21]. Among these alternatives, zirconia-based dental ceramics exhibit enhanced mechanical strength compared to conventional glass-ceramic restorations [22]. Therefore, the patient provided written informed consent to finalize the prosthesis with a zirconia-based ceramic crown.

Dermatologists have previously emphasized the significance of not only excising metal prostheses, but also thoroughly examining and addressing localized infections, including apical lesions and periodontitis [16,20]. As such, in our patient diagnosed with severe peri-implantitis, the implants located in the anterior maxilla were removed and replaced with a temporary crown (Figure 3). However, the chronic purulent apical periodontitis in tooth 27 did not exhibit any improvement, and the tooth was ultimately extracted. Furthermore, the implant located near the maxillary sinus at position 25 displayed no indications of inflammation, leading to the decision to retain it. The extraction of all metallic prosthetic devices affixed to the maxilla and mandible of our patient with ACD was primarily aimed at minimizing the introduction of metal particles into the body. This was achieved by utilizing adequate irrigation, oral suction, regular rinsing, and rubber dams. Subsequently, all prosthetic devices were replaced with provisional crowns, and after placing the zirconia ceramic crowns on the mandible, a precise impression of the abutment teeth in the anterior region of the maxilla was obtained (Figure 4). Ultimately, metal-free prosthetic treatments were achieved for all abutment teeth. However, metal removal replacement therapy did not demonstrate efficacy in all cases of dental metal allergies in our patient, necessitating diligent monitoring following the metal removal (Figure 5). Consequently, it took over two years to ascertain that there were no irregularities present in the temporomandibular joint or oral cavity following the placement of the final prostheses (Figure 6). After prosthetic treatment, a tendency towards improvement was observed in the patient’s periungual ACD (Figure 7). 

In this case, although we lack a comprehensive record of the entire progression, we noted a gradual enhancement in the condition of ACD commencing approximately at the time it was substituted with temporary crowns. Prior studies conducted by dermatologists have indicated that approximately 80% of patients who had allergenic metals removed from their prosthetic devices were classified as “improved” or “significantly improved” post-removal [23]. Upon the patient's arrival at the hospital, a significant duration had elapsed since the placement of the implant and metal prosthetics. Although the underlying occurrence of ACD remains unclear, it has been hypothesized that the removal of suspected allergenic metals from the oral cavity in patients with ACD may diminish the leaching of these metals, thereby facilitating the transition from the elicitation to the sensitization phase of the metal allergy. However, titanium was excluded from the materials evaluated in the patch test, and no potentially allergenic metals were detected in this case. The management of dental metal allergies primarily involves the extraction of suspected metal prosthetic restorations that may act as allergens within the oral cavity, followed by their substitution with non-allergenic materials. As a result, we opted to retain the implant located near the maxillary sinus at position 25, restricting our interventions to the removal of inflammation and the implementation of metal replacement therapy.

It is evident that prosthodontists occupy a pivotal position in this therapeutic approach. Consequently, prosthodontists are integral to both the diagnosis and investigation of dental metal allergies. It is imperative to underscore the need for additional research to elucidate the mechanisms underlying dental metal allergies in ACD and assess the effects of metal replacement therapy.

## Conclusions

In the current case, a patch test was performed on a middle-aged woman diagnosed with ACD whose primary concern was severe peri-implantitis. She had rough eczema-like fingers with cracked nails. The patient showed positive reactions to various dental metals, prompting the removal of the implant associated with peri-implantitis and replacing it with a temporary crown. All metal prosthetic devices were extracted except for the implant located on 25 from the patient’s oral cavity and were replaced with temporary crowns, in addition to the extraction of any other teeth deemed to have a poor prognosis. Ultimately, prosthetic rehabilitation was conducted using zirconia-based ceramic crowns on all abutment teeth, which resulted in a noticeable improvement in the contact dermatitis observed around the patient’s nails. Although this report does not clarify the pathological relationship or underlying mechanisms linking metal allergy to ACD, the alleviation of the metal allergy and ACD symptoms following metal replacement may be beneficial to such patients.
